# Predictions of task using neural modeling

**DOI:** 10.3389/fnrgo.2022.1007673

**Published:** 2022-11-23

**Authors:** Elizabeth L. Fox, Margaret Ugolini, Joseph W. Houpt

**Affiliations:** ^1^Air Force Research Laboratory, Wright-Patterson AFB, Dayton, OH, United States; ^2^Ball Aerospace, Dayton, OH, United States; ^3^Department of Psychology, The University of Texas at San Antonio, San Antonio, TX, United States

**Keywords:** brain-computer interface, task, mental workload, generalizability, EEG

## Abstract

**Introduction:**

A well-designed brain-computer interface (BCI) can make accurate and reliable predictions of a user's state through the passive assessment of their brain activity; in turn, BCI can inform an adaptive system (such as artificial intelligence, or AI) to intelligently and optimally aid the user to maximize the human-machine team (HMT) performance. Various groupings of spectro-temporal neural features have shown to predict the same underlying cognitive state (e.g., workload) but vary in their accuracy to generalize across contexts, experimental manipulations, and beyond a single session. In our work we address an outstanding challenge in neuroergonomic research: we quantify if (how) identified neural features and a chosen modeling approach will generalize to various manipulations defined by the same underlying psychological construct, (multi)task cognitive workload.

**Methods:**

To do this, we train and test 20 different support vector machine (SVM) models, each given a subset of neural features as recommended from previous research or matching the capabilities of commercial devices. We compute each model's accuracy to predict which (monitoring, communications, tracking) and how many (one, two, or three) task(s) were completed simultaneously. Additionally, we investigate machine learning model accuracy to predict task(s) within- vs. between-sessions, all at the individual-level.

**Results:**

Our results indicate gamma activity across all recording locations consistently outperformed all other subsets from the full model. Our work demonstrates that modelers must consider multiple types of manipulations which may each influence a common underlying psychological construct.

**Discussion:**

We offer a novel and practical modeling solution for system designers to predict task through brain activity and suggest next steps in expanding our framework to further contribute to research and development in the neuroergonomics community. Further, we quantified the cost in model accuracy should one choose to deploy our BCI approach using a mobile EEG-systems with fewer electrodes—a practical recommendation from our work.

## 1. Introduction

With continued developments in sensing and analyzing neural activity, there is significant interest in the possibility of passively monitoring mental states, which in turn could be used to adapt the work environment to an operator's needs in real time (e.g., Yuksel et al., 2016; Dehais et al., 2019). One sensor technology, electroencephalography (EEG), holds particular promise as it has high temporal resolution (Kramer, 1991; Smith et al., 2001) and the measured signals correlate relatively highly with task performance (Kramer, 1991; Gevins and Smith, 2000; Kramer and Weber, 2000; Parasuraman and Rizzo, 2006; Gawron, 2008; Andreassi, 2013). Modern machine learning (ML) techniques are critical to extracting the appropriate information from the EEG signal, but unfortunately, a perpetual issue in ML, the trade-off between bias and variance, also limits the ability to deploy adaptive systems based on EEG signal outside of laboratory settings. In particular, the most precise predictions of human performance based on EEG signal are those trained on highly similar data. Human EEG signals are nonstationary in nature, meaning even within a single subject doing the same task, there is “drift” in the measurement over time that can lead to misclassifications by a trained ML system (Fairclough and Lotte, 2020). While techniques for online model adaptation to drift exist, they are complex and computationally expensive (e.g., Li et al., 2021). The lack in generalizability, i.e., the bias of analysis, is much more dire when attempting to predict performance on different sessions or anything about cognitive state when a person performs a distinct task, let alone when a system is applied to a different operator (see Zhou et al., 2021, for a recent review). At the other end of the bias-variance trade-off scale, specific metrics based on power at predetermined spectra and prespecified sensor locations have been proposed to measure general cognitive states such as attention and mental workload. While these measures are not specific to task, time, nor individual, they offer only a crude and noisy, i.e., high variance, picture of the operators cognitive state and hence could lead to frequent misclassifications. The goal of our research reported here is to examine the bias-variance trade-off when using support vector machines (Cortes and Vapnik, 1995; Ben-Hur et al., 2001) to recover information about task and workload from EEG data.

In this work, we focus on multi-tasking performance for two reasons. First, humans either choose to or are required to complete multiple tasks, each typically with an independent goal and level of urgency, both in workplace settings and in their personal lives. Second, mental workload is a major topic of research in human-factors psychology, and hence, there is an extensive literature on predicting multi-tasking performance based on estimated mental workload, including from EEG signals. When taking on additional tasks, numerous multitask demand manipulations can influence mental workload, performance, and neural activity. A few task demand manipulations include: a simultaneous change in the level of difficulty for the entire environment (i.e., all subtasks), the addition (subtraction) of a subtask(s), and the degree of task similarity where subtasks compete for more (less) common resources (Wickens, 1984). Typically, neural predictors of multi-tasking performance involve one manipulation. Different manipulations of task demand, for instance—the latter two examples, may activate different patterns of neural activity; thereby altering which neural indices are most informative to measure mental workload and predict current, or future, performance. Hence, we assessed how the indices of neural activity reliably vary when the entire environment increases in difficulty and how this generalizes to other types of task demands. Specifically, we investigated whether consistent patterns of brain activity may predict two types of task demands, i.e., number of tasks (one, two, or three), and types of attentional resource demands (tracking, communicating, monitoring).

Measurements from a typical EEG system include information across the full range of cognitively relevant frequencies at an extremely high temporal resolution. Neural activity at particular electrodes and frequency bandwidths fluctuate in response to the amount of resources demanded from the task environment to maintain adequate performance and, compared to event related potentials, varies by task type in addition to workload (Ke et al., 2021). Specifically, theta (4–7 Hz), alpha (8–13 Hz), beta (14–25 Hz), and low gamma (25–40 Hz) wave activity consistently vary in correspondence with the level of task demands, and we summarize this related work in the next few paragraphs.

Theta band activity varies with the relative degree of concentration one provides to completing task requirements. As task load increases, theta activity increases (Smith et al., 2001; Holm et al., 2009; Antonenko et al., 2010; Puma et al., 2018). However, both increases and decreases in alpha power have been associated with increased task demands and allocation of effort. Alpha wave activity is associated with subjective relaxation and default mode network activation (Knyazev et al., 2011) and has similarly been shown to have an inverse relationship with task difficulty (e.g., Freeman et al., 1999; Fallahi et al., 2016; Puma et al., 2018). In contrast, some studies have found increased alpha power with increased workload or task difficulty (Zhao et al., 2012; Borghini et al., 2014; Kamzanova et al., 2014; Wisniewski et al., 2017). These contrasting results may be due to differences in task requirements, particularly working memory demands. If increases in alpha power at a particular brain region are interpreted as having a suppressive effect, then one would expect alpha power increases in brain areas that would process irrelevant or distracting information (for example, visual cortex during a challenging listening task).

There are also conflicting results in the literature regarding the relationship between beta wave activity and engagement. Some research has found that as task demands increase, subjective engagement and beta activity increases (Pope et al., 1995; Prinzel III et al., 2003), while others find the opposite. These changes in beta wave activity may stem from electromyographic (EMG) activity, like eye or muscle movement, rather than neurological activity (Spydell et al., 1979; Spydell and Sheer, 1982). Regardless, EEG beta activity may still be a proxy for predicting performance: a high degree of mental workload may correlate with higher or lower eye or muscle movements depending on task and response demands, leading to lower efficiency.

Pope et al. (1995) combined alpha and beta power, along with theta activity into a single score that correlates with task demands and performance (Pope et al., 1995; Prinzel III et al., 2003). They posited that a rise in cognitive demands leads to a larger increase in beta activity than the sum of alpha and theta activity (i.e., Index = beta/(alpha + theta); Brookings et al., 1996).

Although alpha, beta, and theta have received the most attention in the literature, fluctuations in gamma power may also track workload, task demands, and task engagement^1^. An increase in gamma power is associated with attention and perception, specifically related to visual information (Müller et al., 2000). Gamma power increases are also associated with higher subjective ratings of workload and error rates (Choi et al., 2018), can be used to distinguish workload even while the individual is walking (Tremmel et al., 2019), and can be used to differentiate task type (Ke et al., 2021).

Some authors have suggested that multi-tasking elicits more activity in particular brain areas; this activation systematically increases as more tasks are added and is not evidence of increased difficulty in a single-task environment (D'esposito et al., 1995; Adcock et al., 2000; Cudmore et al., 2000; Sigman and Dehaene, 2008). Specifically, D'esposito et al. (1995) found increases in unique neural activity located around the dorsolateral prefrontal cortex (DLPFC) when completing two, generally unrelated tasks, simultaneously. Therefore, they characterize the DLPFC as a primary source for maintaining multiple tasks and filtering relevant, vs. distractor, information in a multi-tasking environment.

With respect to EEG, some authors have recommended specific electrodes for measuring workload. For example, frontal midline (Fz) theta activity increases and parietal midline (Pz) alpha activity decreases as the task difficulty increases in a multi-tasking environment relative to a passive watching condition (Gevins and Smith, 2003). Cannon et al. (2010) recommend a standard set of electrode and frequency pairs for measurement and specific sites for EEG electrodes that characterize task demands for complex cognitive tasks including: EOG data (theta), F7 (alpha, theta), Fz (alpha, theta), Pz (alpha, theta), T5 (alpha, beta), and O2 (alpha, beta). Note that these features tend to be evaluated for the full task environment. Each subtask component alone may encompass a more task-specific list of features, depending on the nature of the subtask component.

Some research has suggested an over-additive effect in multitasking environments such that higher theta and beta band activity and lower alpha activity occur (Adcock et al., 2000). Others suggested when multitasking demands superseded an operator's mental limit, they transitioned to the “overload region”, where specific features of neural activity declined (Just et al., 2001). Subsequent research consistently demonstrated the increases and decreases in neural activity over time as hypothesized by previous research. However, there was not a reliable correlation between changes in neural activity and observed changes in performance (Serrien et al., 2004).

The generalizability of multi-task specific brain areas is questionable; some demanding multi-task combinations that elicit task performance decrements and overall neural activity increases/decreases in specific bandwidths often associated with mental workload do not illustrate the corresponding differences in DLPFC activation. Rather, the neural activity of these multitask combinations that will change in accordance with task demands is representative of the electrode-bandwidth activity evident when each subtask is performed in isolation at various levels of difficulty (e.g., Brookings et al., 1996; Ke et al., 2014).

Understanding of the association between activity in individual frequency bands and task load and type can be combined with more data-driven machine learning methods to better classify task load and type. The 2021 Neuroergonomics Grand Challenge: Passive BCI Hackathon is a recent large scale effort to leverage EEG data to classify workload. All teams participating in the challenge used the same dataset: EEG data from 15 participants while they completed various combinations of subtasks at 3 levels of difficulty, collected across 3 days. Challenge teams were asked to develop an algorithm that decodes workload level. The algorithm could be retuned on an individual subject basis, but needed to classify data from the third, unseen session. The winning entry to the challenge (Singh et al., 2021) used support vector machine classification. The selection of EEG channels was based on a maximal distance between the three workload categories in a Riemannian space representation of the data, considering only the theta and beta EEG bands. Classification performance was 54% averaged across all participants. The results from this challenge provide further evidence that a machine learning method, combined with the field's existing understanding of which aspects of EEG activity reflect workload and task demand changes, can be useful for a BCI system.

Extracting information from large, multivariate datasets such as EEG signals requires care, particularly due to the aforementioned bias-variance trade-offs. Support vector machines (SVM) are one way to pull together multiple characteristics of a neural signal to maximally differentiate between multiple datasets, collected from different environments, and hence allow us to quantify the classifier performance at different levels of bias-variance trade-offs. We used SVMs to investigate the stability in the relationship between different components (i.e., bandwidths, electrodes) of neural activity (e.g., frontal-theta) as we manipulated task demands. Generally, our research advanced the field in three ways:

1. Two manipulations to task demands: the number of tasks and the degree of competition for common resources (dual-tasks);
2. Comparison in the accuracy of 10 electrode-frequency bandwidth subsets to inform machine-learning model predictions at the individual-level;
3. Assessment of model generalizability through the comparison of within- and between-session prediction accuracy.

We subset the full set of electrode-bandwidth components (320) and those outlined as significant predictors of task demands in previous research. In total, we tested the full electrode-bandwidth set, and 10 subsets; each is outlined in Table 1. We tested the degree that neural activity in each subset informed a machine-learning model to predict 1 of 7 single- and multi-task condition categories of varying demands. We assessed the machine learning models' predictive accuracy of task condition, where task conditions consisted of two types of demand manipulations: numbers of tasks and competition for common resources. We then tested the most effective model using subsets of electrodes that correspond to a number of popular mobile EEG headsets to better estimate how our model performance may be altered in a real-world scenario. Next, we outline previous studies and their conclusions about neural activity variations in response to changes in task demands.

**Table 1:** The full model and 10 electrode-bandwidth subsets.

**Set #**	**# of Features**	**Electrode-bandwidth pairs**	**Previous suggestion of indices**
1	320	64-channels (δ, θ, α, β, γ)	Casson, 2014 (19-channels)
2	128	64-channels (θ, α)	Borghini et al., 2014
3	64	64-channels (δ)	
4	64	64-channels (θ)	Antonenko et al., 2010
5	64	64-channels (α)	Smith et al., 2001
6	64	64-channels (β)	Prinzel et al., 2001
7	64	64-channels (γ)	part of Choi et al., 2018
8	64	64-channels ($\frac{\beta }{\alpha +\theta }$)	Pope et al., 1995; Nuamah et al., 2017
9	10	(F7,Fz,Pz)(α, θ), (P7,02)(α, β)	Cannon et al., 2010
10	5	Pz (δ, θ, α, β, γ)	Hogervorst et al., 2014
11	2	Fz (θ), Pz (α)	Gevins and Smith, 2003

The set of electrodes directly corresponds to those provided for the BioSemi-64 EEG system; bandwidths correspond to a specified frequency range, i.e., delta (δ): 2 − 4 Hz, theta (θ): 4 − 7 Hz, alpha (α): 8 − 14 Hz, beta (β): 14 − 25 Hz, and gamma (γ): 25 − 40 Hz.

Previous researchers subset EEG data to specific electrode and frequency pairs and found a priori assumptions limit model reliability and specificity. Specifically, models suffer when they assumed the types of neural activity that were informative to assist in operator support efforts (e.g., adaptive automation, user enhancement). We compared hypotheses of bandwidths and electrodes, as these are highly disputed and relatively unexplored in manipulating number of, and types of, multitask combinations. Alternatively, the 2021 Neuroegonomics Grand Challenge represents a more data driven approach to creating machine learning models of workload. The dataset used in this challenge emphasized classification based on task difficulty (i.e., workload) and not task type (i.e., did the task involve communication? Visual tracking? etc.). Further, these data were cleaned using an offline classification algorithm (IC Label) that would be prohibitive for use in real-time BCI applications. In the present study, we adopted an exploratory machine learning approach to produce a relatively unconstrained model that could take advantage of the abundance of EEG data, that was minimally cleaned and processed. Here the model chose which electrodes, and the bandwidths within electrodes, best captured the differences between task demands to accurately classify task demands and, in turn, predict performance; we created models at the individual-level.

### 1.1. Decoding

Our goal was to use neural activity to establish a model that could delineate unique patterns of brain activity amongst multiple levels of task demands and task types. We tested this model by providing the model new data, from the same participant, and assessing the model's accuracy in correctly identifying the source of task demands.

Multivariate pattern analysis (MVPA) is a machine learning approach to analyze neuroimaging data that takes into account the relationship between multiple variables (e.g., channels of the EEG) instead of treating them each as independent variables measured in relative activation strength (power). Lotte et al. (2007) discuss the strengths and weaknesses of five machine learning approaches that are most useful for brain-computer interface (BCI); they suggest a combination of classifiers is most efficient. However, they describe support vector machines (SVMs) as the leading stand-alone classifier. SVMs are (1) highly generalizable: accommodate to non-stationarity and variability through normalization, (2) simplistic in nature: require minimal parameter specifications, (3) insensitive to overtraining, and (4) robust against the “curse of dimensionality.” Other classifiers are faster to execute than SVMs, but the computational speed of an SVM model is still efficient enough for real-time adaptive automation or intervention (Lotte et al., 2007).

Our approach extends current research by investigating the sensitivity and generalizability of machine learning models in a few ways. (1) Typical decoding of neural data is limited to a binary choice such as high and low cognitive activity (e.g., Baldwin and Penaranda, 2012), although in recent years the decoding of more categories has become slightly more common (for example, see the 2021 Neuroergonomics Grand Challenge); we developed a model that each distinguished among 7 conditions. (2) Difficulty in multi-tasking studies is primarily manipulated by increasing the demands of the full environment, equally balanced between subtasks (e.g., Cannon et al., 2010), or if difficulty is manipulated by adding additional task types there is no effort to identify when each task is being completed (Neuroergonomics Grand Challenge, 2021); we manipulated task difficulty in two ways: the nature of and number of the subtask components and worked to classify both of these things. (3) We estimated the decision boundaries of each model, for each participant; the decision boundaries were set to maximally differentiate the participant's neural activity in each of the different task demand levels. In summary, we developed multiple, individual-level classification models, referred to as a classifier, where each was specific to a participant to predict the type of demand manipulation (i.e., the number of subtasks, the types of subtasks).

The first step of decoding was to use the algorithm of choice (e.g., SVM) to place decision boundaries in a higher-dimensional space that best separated the patterns of brain activity that corresponded to each experimental condition of interest. Following the recommendations of Brownlee (2019), we fit the model using a random sample subset (67%) of data, referred to as the training data. In a recent review, Nalepa and Kawulok (2019) thoroughly discussed the extensive body of research on selecting training sets SVMs and stated that random sampling is simplistic and easy to implement, does not depend on time, and is a practical choice for real-time BCI. The drawback of random sampling is the propensity to require laborious outlier and noise reduction before randomly selecting the training set.

After fitting the model to a random subset of neural data, the model was subsequently evaluated using the remaining third of the data. We assessed classifier performance relative to chance performance. We defined chance accuracy as that which would be predicted by uniform random labeling across possible category labels. For example, we provided a classifier seven categories whereby decision bounds were set to maximally separate neural data between each; therefore, the level of chance for a classifier with seven categories was 1/7 or 14.3%. If the classifier performed higher than predicted by chance on the new set of data, there was evidence that the classifier generalized the learned associations to labeling new brain response data patterns. We tested the classifier with the data that was partitioned out before training the model (33%), such that the category label was unknown to the classifier. To assess the accuracy of the classifier, we recorded the category that the algorithm identified as appropriate for the testing data, given the model fit to the training data, and compared it to the true category label. Therefore, to assess classifier accuracy, it was necessary to have access to the true category label for all data used for testing purposes. We recorded the accuracy of the classifier to classify data correctly or incorrectly.

## 2. Methods

### 2.1. Participants

Twenty participants (18 Caucasian; 2 Asian), 12 female (Age: *M* = 25, range = [18, 34]) and 8 male (Age: *M* = 25.6, range = [18, 31]), completed three sessions. Each session lasted no longer than 2 h (Session 1) or 3 h (Session 2 and 3) for any participant. Participants were compensated $10/h after each session ($80 in total). The duration of the full study ranged from 3 to 10 days depending on the availability of the participant and laboratory space; each session was separated by 1–3 days. All participants' primary language was English and all indicated they were fluent in their ability to read, write, and comprehend the English language. All participants had normal color vision and normal (20/20) or corrected to normal (glasses/contacts) visual acuity.

### 2.2. AF-MATB description

Participants performed the Multiple-Attribute Task Battery (MAT-B; developed by Comstock and Arnegard, 1992), later adapted by the Air Force (AF-MATB; Miller et al., 2014). In the current experiment, we utilized three subtasks while “blacking-out” the remaining tasks. Below we briefly provide details pertaining to the goal and demands of each subtask. For those interested in more details about the subtasks or performance, participant performance was modeled using an established measure, Multitasking Throughput (MT), and published in Fox et al. (2021).

#### 2.2.1. Monitoring task (M)

**Figure 1:**
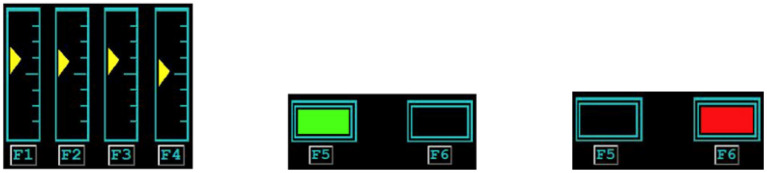
**Left**: The Gauges used in the Monitoring (M) subtask of AF-MATB. **Middle**: The Lights used in the Monitoring (M) subtask of AF-MATB, both properly functioning (Left = Green, Right = Black). **Right**: The Lights used in the Monitoring (M) subtask of AF-MATB, both signaling a malfunction (Left = Black, Right = Red).

Participants had to identify and “fix” malfunctions that occurred in four gauges and two lights (Figure 1) using keyboard responses (i.e., F1-F6). The participant watched each gauge and judged the extent to which each slider fluctuated above and below the center dash, a slightly longer dashed line indicated the center. A “malfunction” occurred when the slider moved outside of an acceptable range: one tick mark above or below the center dash. During the malfunction the slider would alternate between the malfunctioning and normal range of tick marks. If the participant identified and corrected the malfunction within the allowed amount of time (10 s) the slider returned to the center of the gauge and began alternating above and below the center range once again. A yellow bar at the bottom of the gauge was presented to signal to the participant that their response was detected and correct.

The participant had to monitor two lights. The light on the left was green, “ON,” and the light on the right was black, “OFF,” during normal operations. A malfunction occurred when either the left light turned black, “OFF”, or the right light turned red, “ERROR.”

#### 2.2.2. Communications task (C)

**Figure 2:**
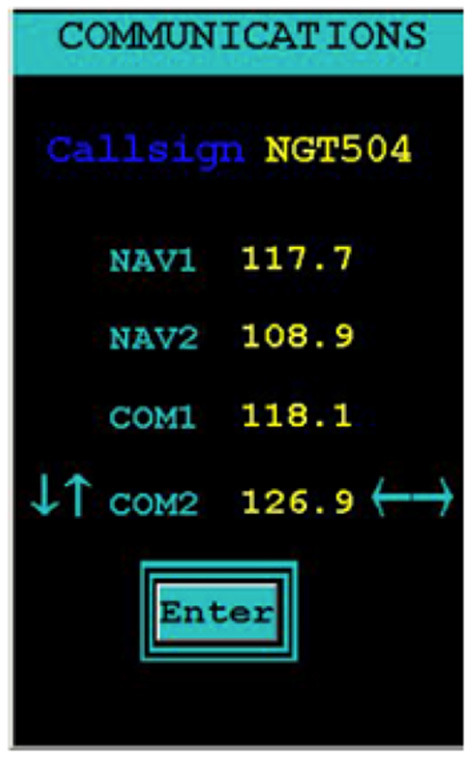
The Communications (C) subtask in the AF-MATB environment.

Participants listened for an auditory transmission that first announced their assigned call sign, “NGT504,” followed by a particular radio channel (4 possible channels) and a specific frequency (illustrated in Figure 2). The call sign, “NGT504,” was fixed for all participants and sessions; it was visible at all times. Participants used their left hand to press the keyboard arrow buttons and navigate through channels (up/down arrows) and frequencies (left/right arrows). Participants again used their left hand to press the enter key and submit a response; the visual “Enter” flashed green to signal to the participant that their response was submitted, regardless of whether it was a correct or incorrect response. Two types of events could occur: True communications events (TC) and false communications events (FC). Each type of event demanded a particular participant response. TC events occurred when the audio transmission addressed the participant's designated “call sign.”

#### 2.2.3. Tracking task (T)

**Figure 3:**
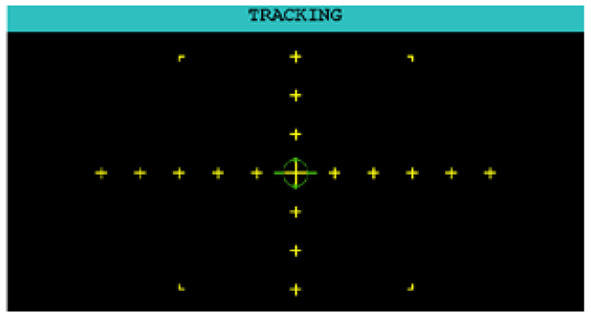
The Tracking (T) subtask in the AF-MATB environment.

The participant used a joystick to control the movement of a green, circular reticle (illustrated in Figure 3). The objective was to steer the reticle as close to the center yellow crosshair as possible. The AF-MATB software included three default levels of difficulty where each increase in difficulty corresponded to more frequent changes in direction and faster movement of the reticle. For the analyzes reported here, we utilized the “Medium” level of difficulty.

### 2.3. Experimental sessions

#### 2.3.1. Training session

The purpose of the first experimental session (a maximum of 2-h) was to train and familiarize participants with each single-task alone, dual-task pair, and the triple-task combination. We pseudo-randomized 3-min trials for each of the seven trial types (T-alone, C-alone, M-alone, T+C, T+M, C+M, T+C+M); this set of seven trials was repeated 3 times for each participant split with an optional break between each set (21 trials in total). All task components were set to a moderate level of difficulty, see Fox et al. (2021) for specific event details.

#### 2.3.2. EEG sessions

The remaining two sessions had a pseudo-randomized, factorial design of single- to triple-task combinations. For each participant, neural data were collected across 2 days, each containing thirteen (each 6 min) trial types. Task demands for a given task could vary from low (indicated by a subtext of 1; e.g., T1), moderate (indicated by a subtext of 2; e.g., T2), or high (indicated by a subtext of 3; e.g., T3). For the modeling reported in this paper, we only use data from the fixed ‘moderate' level of difficulty. Therefore, trial types included each factorial combination of single- to triple-tasks at a moderate level of difficulty (i.e., T2, C2, M2, T2C2, T2M2, C2M2, T2C2M2). Participants used a keyboard and joystick to respond; audio transmissions came through external speakers.

### 2.4. Materials

#### 2.4.1. Hardware

We required 2GB of RAM, 2GHz dual-core processor, a 15-inch monitor, a keyboard, a mouse, a joystick, and speakers to run the AF-MATB software. These were requirements for the tasks to function properly as intended by the software development team. A keyboard and joystick were necessary for the participant to respond to experimental stimuli and we, as the experimenter, used a mouse to press the start button to begin the session. Speakers allowed the participant to hear the audio transmissions of the Communications task. To protect our EEG recording from exposure to excessive noise, we collected all data inside a Faraday cage. The experimenter sat outside of the Faraday cage, and monitored the subject directly *via* a small webcam placed in front of them. The experimenter also viewed the EEG data and the participant monitor in real-time for the duration of the experiment.

**Figure 4:**
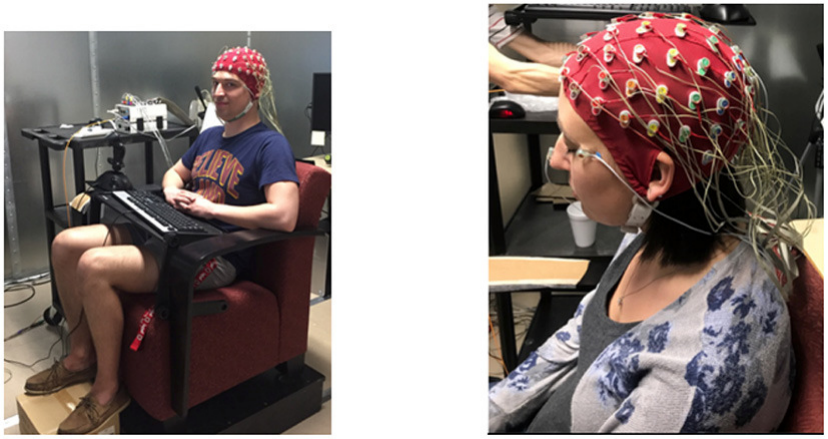
**Left**: An example image of our post-training laboratory set-up. Here we collected EEG data during the last two sessions (post-training) of the experiment. **Right**: An example of the 64-electrode BioSemi system on a human subject using an ECI and external mastoid and EOG electrodes.

The EEG signals were recorded in two experimental session by a 64 Ag-AgCl pintype active electrodes (ActiveTwo, BioSemi) mounted on an elastic cap (ECI) according to the extended 10–20 system, and from two additional electrodes placed on the right/left mastoids, and an electrode on the tip of the nose (example in Figure 4). Eye movement and blinks were monitored using EOG electrodes. The EEG and EOG were sampled at 1,024 Hz with 24-bit resolution and an input range from −262 to +262 mV/bit. EEG data were saved and processed offline.

#### 2.4.2. Software

The AF-MATB software we used required Windows 7, MATLAB Compiler Runtime 7.8, and Microsoft Office 2007 (Miller et al., 2014). We used a combination of Python and R for processing the data; each provided unique strengths to facilitate processing models of performance data, neural data, and the implementation of machine learning algorithms. We used ActiView to monitor participant's brain activity during the experiment in real-time. Additional software, provided by National Instruments (NI-DAQmx and NI-MAX), were necessary for the AF-MATB to pass triggers, i.e., information about what events happened in the participant's task environment, to the BioSemi USB2 receiver.

### 2.5. Model evaluation

We established 3 criteria to evaluate classifier performance and chose the “best” parameters and model customization, for each participant separately. We defined our criteria with the intention to adequately capture the characteristics we found most valuable in a decoding algorithm: consistency, accuracy, and low propensity to over fit. A rigorous and sophisticated approach where we compared numerous algorithms and parameter spaces is desirable and would likely yield interesting findings; however, the full exploration of modeling alternatives (e.g., k-means clustering, linear discriminant analysis, etc.) is beyond the scope of this paper.

Consistency was the degree to which the accuracy of the model stayed the same when trained on different random subsets that were sampled from the same overarching data set. A high degree of consistency indicated the algorithm was resilient to sporadic noise that is often littered throughout raw EEG data. Accuracy was the degree to which the model identified data as coming from the category that corresponds to the true (known) category label. We assessed the accuracy of the model in categorizing the data in which it was trained on, and a subset of data that the model was “blind” to during the training phase (33%). If we over-fit the model to a subset of data, it decreases the ability for the trained classifier to generalize and make accurate category predictions using new data. We determined the propensity for a model to overfit to the training data by calculating the change in accuracy when testing the algorithm on the training data and the testing data set. If accuracy substantially decreased when the model was used to predict the testing data, as opposed to the training data, then we concluded the algorithm, for our purposes, had a higher propensity to overfit. Based on these criteria, we chose to use SVM for analyzing these data. SVMs accommodate a high volume of features; we had 320 features. Additionally, SVM models typically performed well (i.e., high accuracy) in classifying data across multiple days in a complex multi-tasking environment, compared to alternative machine learning models (Christensen et al., 2012).

#### 2.5.1. Model prediction

We split the data in two parts: training and testing data. We used cross-validation such that the model was blind to the test data. We computed model accuracy for each classifier and individual. The classifier modeled the bounds of 7 categories: T, C, M, T+C, T+M, C+M, T+C+M; chance performance for this model was 14.3%. We calculated the accuracy of the model to predict data across multiple levels of generalizability (Table 2). The training data were the subset of data that were used to estimate the model bounds. The within-day testing data were a subset not used for training but were collected on the same day. Between-day testing were a subset of neural data (same participant and task demands) collected 1–3 days before or after the training set (Note: “before” or “after” was dependent on which data set, Day 1 or Day 2, the model was trained on). We constructed and tested models for each participant, for each of the two model types, at each level of generalization. In Table 2 we provide a summary of the 4 ways we defined and tested each model. We trained and tested a separate set of models for the full model and 11 subsets of electrode-bandwidth features, shown in Table 1. We swapped the data set used for training and testing such that Training: Day 2 (67%), Within Day: Day 2 (33%), Between Day: Day 1 (33%) and repeated these analyzes a second time.

**Table 2:** Summary of individuals' classifier accuracy to predict number of tasks (single-task, dual-task, triple-task).

	**Training data (67%)**		**Testing data (33%)**	
**Index**	**Level**	**Day**	**Level**	**Day**
1	Individual	Day 1	Individual	Day 1
2	Individual	Day 2	Individual	Day 2
3	Individual	Day 1	Individual	Day 2
4	Individual	Day 2	Individual	Day 1

### 2.6. Model parameters and specifications

We used cross-validation to learn the best parameters for our data. The hyperparameters of the model include a class weight, kernel, and two corresponding to the trade off between model specificity and generalizability (denoted as C and gamma). To choose the fixed values of these parameters, we randomly selected a single participant's data, normalized their data (mean = 0, variance = 1) and did a parameter sweep across a range of values, for an SVM model to find the bounds between numbers of tasks. As a result, we selected C=100.0 and gamma=0.001. For all models, we used a 5-fold cross validation method to obtain model accuracy.

We tested whether to restrict our model to only fitting linear functions. A linear model typically was implemented as a single support vector that distinguishes the respective data from data of all other categories; this is called a “one-vs-the-rest” approach. We tested a linear model against a “one-against-one” approach (Knerr et al., 1990) that allowed for nonlinear modeling between categories. In a nonlinear model, each support vector was trained on data between two categories; the number of constructed support vectors was Nc * (Nc-1) /2 where Nc equals the number of categories.

Using a subset of category predictions (predict number of tasks: 1, 2, or 3), we computed the accuracy (% correct) of the classification model. We examined the additional benefit of using a nonlinear SVM function for our model over and above linear SVMs. In sum, we found the nonlinear function, i.e., the radial basis function (RBF), to outperform a model based on linear SVMs to accurately predict training data, within-day data withheld from training for both multi-class decisions. The nonlinear model was also superior for classifying the number of tasks between-days; however, the models performed equally well at classifying the type of tasks, a 3-class decision (Table 3). Furthermore, we fixed the best RBF parameter values when we trained all participants' SVM models.

**Table 3:** Linear vs. nonlinear model comparisons for one participant's data to predict the number of tasks.

		**Training data**		**Within-Day**		**Between-Day**	
**Training**	**Chance**	**Linear**	**RBF**	**Linear**	**RBF**	**Linear**	**RBF**
Day 1	33.3%	89.6%	**100.0%**	78.1%	**86.9%**	47.7%	**52.4%**
Day 2	33.3%	92.6%	**100.0%**	82.5%	**89.9%**	46.1%	**51.2%**

We compared a linear and nonlinear support vector machine to predict the number of tasks on the training data, data held-out from the same day (“Within-Day”), and data that was collected on a different day (“Between-Day”). Models predicted one of three categories (chance rate = 33%).

Bold values indicate the radial basis function (RBF) model outperformed the linear model.

### 2.7. Data cleaning

We modeled neural activity at the individual-level. Therefore, we only kept the participants where neural activity was appropriately collected and stored for all task conditions (T, C, M, T+C, T+M, C+M, and T+C+M) across both Day 1 and Day 2. Due to incomplete or corrupt neural recordings, we eliminated 4 of the 20 participants (*N* = 16).

### 2.8. Pre-processing

Files for each individual, condition, and session were processed separately. Pre-processing was completed using mne (Gramfort et al., 2014) in Python. Raw data were loaded and referenced to the average of the two mastoids. Data were then bandpass filtered from 1 to 40 Hz using a firwin filter. The first 10 s of each run was removed to exclude noise that commonly occurred at the onset of the experiment. Data were then segmented into partially overlapping windows—each window as 2 s in length with a 1 s shift. Average power was then calculated for each electrode using the Welch method with a Hanning window and density scaling in the following bandwidths: delta (2–4 Hz), theta (4–7 Hz), alpha (8–14 Hz), beta (14–25 Hz), and gamma (25–40 Hz). Mean power spectral density (PSD) was calculated for each bandwidth.

## 3. Results

We trained and tested 704 models (N x F x I): N = total number of subjects (16), F = number of input neural feature sets (11: Table 1), and I = model generalizability index (4: Table 2). In Table 4 we report the average percent correct between using Day 1 or Day 2 for training the model. We computed the average predictive accuracy of the individualized SVM models to classify data at multiple levels of generalizability: 1. data used to train the model, 2. testing data collected on the same day, and 3. testing data collected on a separate day, where “testing data” is a portion of data set aside when training the ML model.

**Table 4:** Summary of individuals' classifier accuracy to predict type of task(s): T, C, M, T+C, T+M, C+M, and T+C+M.

			**Train**	**Test**	
**Set #**	**# of Features**	**Channels**		**Within-Day**	**Between-Day**
1	360	64-Channels (δ, θ, α, β, γ)	**99.32%**	**84.49%**	**32.01%**
2	128	64-Channels (θ, α)	91.89%	61.54%	26.16%
3	64	64-Channels (δ)	67.10%	49.50%	21.33%
4	64	64-Channels (θ)	69.10%	49.25%	21.43%
5	64	64-Channels (α)	69.78%	53.55%	25.24%
6	64	64-Channels (β)	82.53%	69.40%	25.92%
7	64	64-Channels (γ)	89.66%	82.77%	27.63%
8	64	64-Channels ($\frac{\beta }{\alpha +\theta }$)	61.82%	19.78%	15.01%
9	10	(F7, Fz, Pz)(α, θ), (P7, O2)(α, β)	36.55%	33.46%	23.89%
10	5	Fz(δ, θ, α, β, γ)	27.68%	25.63%	19.59%
11	2	Fz(θ), Pz(α)	22.52%	21.33%	19.22%

Average percent accuracy of individuals' classifiers predict type of task(s): T, C, M, T+C, T+M, C+M, T+C+M for each feature set outlined in Table 1. Chance performance was 1 of 7 (14.3%). Same day refers to the average score across both classifier Index 1 and 2 (Table 2) such that Day 1 or Day 2 was both used for training and testing the model. Different day refers to the average score across both classifiers Index 3 and 4 (Table 2) such that Day 1 (Day 2) was used for training in Index 3 (Index 4) and Day 2 (Day 1) was used for testing.

Bold values indicate the accuracy of the full model.

**Figure 5:**
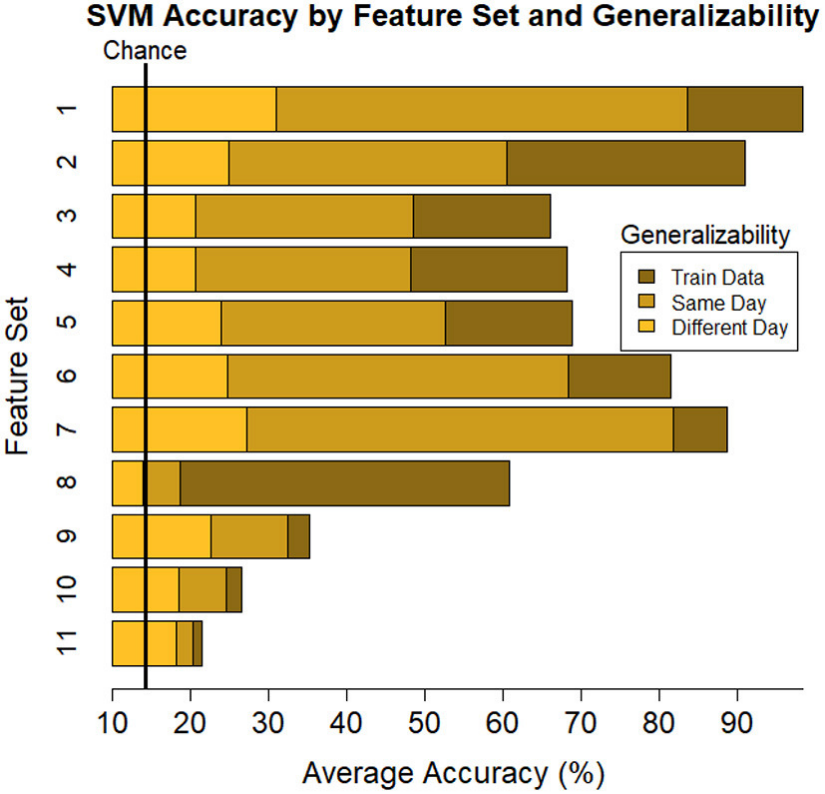
Plot of summary of individuals' classifier accuracy to predict type of task(s): T, C, M, T+C, T+M, C+M, T+C+M, for each feature subset outlined in Table 1. The number of features decreases from top (Set #1) to bottom (Set #11) in a nonlinear fashion, see Table 4 *# of features*. Numeric averages are provided in Table 4. Chance performance was 1 of 7 (14.3%). Set number refers to the electrode and bandwidth pairs included in the feature set used for classification. 1) All electrodes (δ, θ, α, β, γ), 2) All electrodes (α, θ), 3) All electrodes (δ), 4) All electrodes (θ), 5) All electrodes (α), 6) All electrodes (β), 7) All electrodes (γ), 8) All electrodes (α/(β+θ)), 9) F7(α, θ), Fz(α, θ), Pz(α, θ), P7(α, β), O2(α, β), 10) Pz (δ, θ, α, β, γ), 11) Fz(θ), Pz(α). Same day refers to the average score across both classifier Index 1 and 2 (Table 2) such that Day 1 or Day 2 was both used for training and testing the model. Different day refers to the average score across both classifiers Index 3 and 4 (Table 2). In model Index 3, Day 1 was used for training and Day 2 was used for testing. In model Index 4, Day 2 was used for training and Day 1 was used for testing.

Average train and test accuracy for categorizing training data, data from the same day, and data from a different day are given in Table 4 and shown in Figure 5. “Train” results is the average accuracy of every model to predict the same data that was used to train it (averaged across individual (16) and Index 1–4 for each subset of features (11), respectively; *n* = 704). As expected, these predictions always yielded the highest accuracy, *M* = 65.24, [22.49, 99.33], *SE* = [0.21, 3.88]. Same day is the average accuracy of each model to predict data from the same individual collected on the same day as the data used to train the model, but the model was “blind” to these data when training, i.e., these data were left out of the training phase (*n* = 352). As expected, these predictions were lower than the Train predictions, but higher than the Different day predictions, *M* = 50.06, [19.78, 84.49], *SE* = [0.39, 1.78]. Finally, Different day is the average accuracy of each model to predict data from the same individual collected on a different day as the data used to train the model (*n* = 352), and, as expected, these predictions were the lowest among our comparisons, *M* = 23.44, [15.01, 31.94], *SE* = [0.29, 1.33]. Nonetheless, accuracy was still above chance among each of the feature sets, with the exception of Set 8 where the model fit to all electrodes “workload index” (β/(α+θ) consistently resulted in around chance level (14.3%) performance when testing its generalizability to predict task on 1 day when the data used to train the model were collected on a separate day, a within-subject model.

### 3.1. Exploratory analyzes

**Figure 6:**
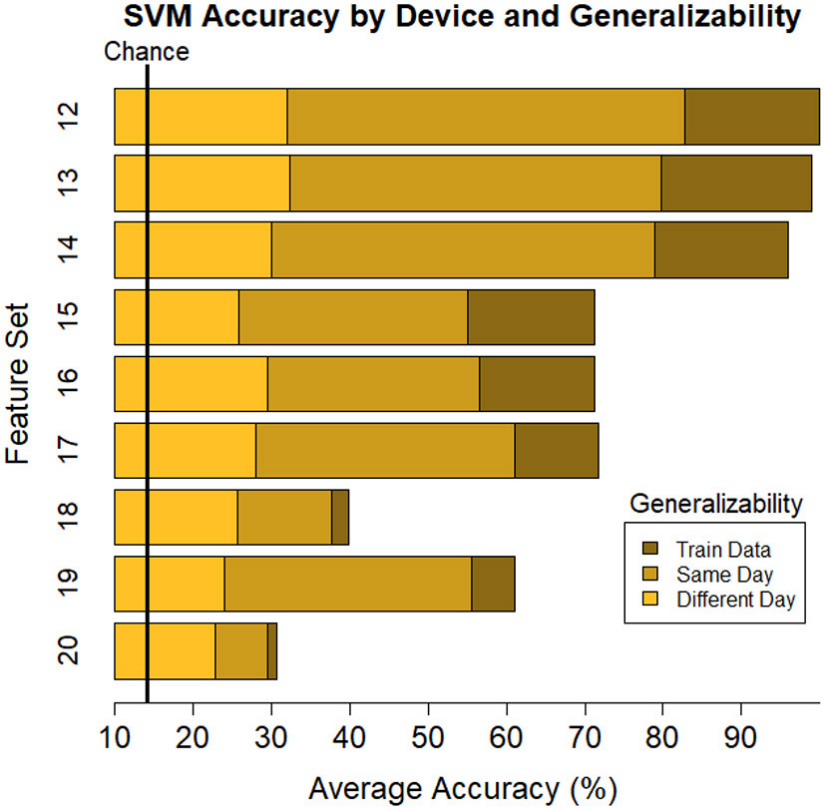
Plot of summary of individuals' classifier accuracy to predict type of task(s): T, C, M, T+C, T+M, C+M, T+C+M, for each feature subset outlined in Table 5. Numeric averages are provided in Table 5. The number of features decreases from top (Set #12) to bottom (Set #20) in a nonlinear fashion, see Table 5 *# of features*. Chance performance was 1 of 7 (14.3%). Set number refers to the electrode and bandwidth pairs included in the feature set used for classification. Same day refers to the average score across both classifier Index 1 and 2 (Table 2) such that Day 1 or Day 2 was both used for training and testing the model. Different day refers to the average score across both classifiers Index 3 and 4 (Table 2). In model Index 3, Day 1 was used for training and Day 2 was used for testing. In model Index 4, Day 2 was used for training and Day 1 was used for testing.

The ultimate goal of neural models of task type and load is real-world applicability. However, moving out of the lab requires the use of mobile equipment, which is often associated with fewer electrodes which often leads to sparser scalp coverage. As an exploratory next step, we quantified the cost in model accuracy should one choose to deploy our BCI approach using mobile EEG-systems with fewer electrodes. We created subsets of features to include electrodes which correspond to state-of-the-art commercial systems and assessed models' accuracy across all bandwidths (delta, theta, alpha, beta, gamma). Mobile systems were selected from recent literature in BMI and workload/task type classification. These systems include the Insight 2.0, Epoc + and Epoc Flex, all from Emotiv, and the Neurosky MindWave (e.g., LaRocco et al., 2020), the Cognionics Quick Cap 20 (e.g., Marini et al., 2019), Neuroelectrics Enobio 8 (e.g., Somon et al., 2022), DSI VR 300 (e.g., Kim et al., 2021), interAxon Muse (e.g., Arsalan et al., 2019), Neurable Enten (e.g., Alcaide et al., 2021), and cEEGrid (e.g., Bleichner and Debener, 2017; Somon et al., 2022). Table 5 depicts each mobile system's electrode montage, reporting the closest possible electrode within the 10–20 system when there was no one-to-one correspondence, and the average percent correct. Our original data set, collected using a 10–20 Biosemi system with 64 electrodes, is provided as set 1 for comparison purposes. We illustrate these results in Figure 6. We computed the average predictive accuracy of the individualized SVM models to classify data at multiple levels of generalizability: 1. data used to train the model, *M* = 70.19, [29.73, 98.95], *SE* = [0.26, 1.23], *n* = 576 2. testing data collected on the same day, *M* = 58.66, [28.57, 81.78], *SE* = [0.75, 1.95], *n* = 288 and 3. testing data collected on a separate day, *M* = 26.87, [21.87, 31.38], *SE* = [0.65, 1.19], *n* = 288.

**Table 5:** Summary of individuals' classifiers to predict task type using subsets corresponding to commercial EEG systems.

				**Train**	**Test**	
**Set #**	**# of Features**	**System**	**Montage**		**Within-Day**	**Between-Day**
1	360	BioSemi ActiveTwo		**99.32%**	**84.49%**	**32.01%**
			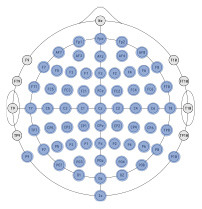			
12	160	Emotiv Epoc Flex		98.95%	81.78%	31.32%
			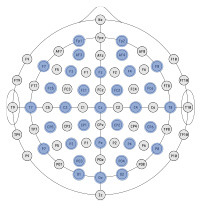			
13	95	Cognionics Quick Cap 20		97.91%	78.77%	31.38%
			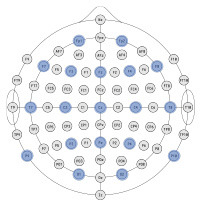			
14	70	Emotiv Epoc +		94.93%	77.92%	29.04%
			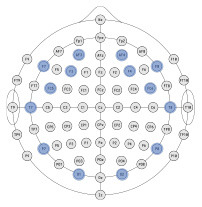			
15	40	Neuroelectrics Enobio 8		70.30%	54.08%	24.93%
			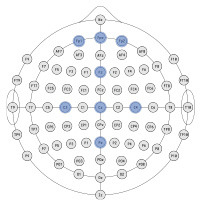			
16	35	DSI VR 300		70.18%	55.54%	28.59%
			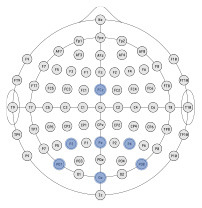			
17	25	Emotiv Insight 2.0		70.69%	60.01%	27.08%
			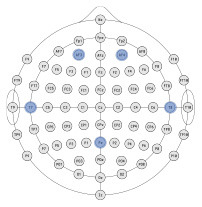			
18	20	interAxon Muse		38.92%	36.71%	24.80%
			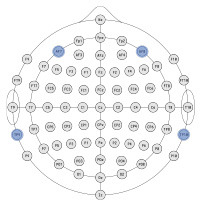			
19	10	cEEGrid/ Neurable Enten		60.11%	54.55%	23.00%
			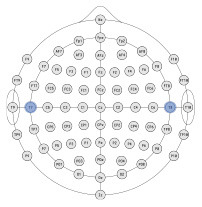			
20	5	NeuroSky		29.73%	28.57%	21.87%
			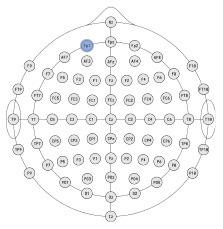			

Average percent accuracy of individuals' classifiers predict type of task(s): T, C, M, T+C, T+M, C+M, T+C+M for each feature set of commercial EEG systems. Chance performance was 1 of 7 (14.3%). Same day refers to the average score across both classifier Index 1 and 2 (Table 2) such that Day 1 or Day 2 was both used for training and testing the model. Different day refers to the average score across both classifiers Index 3 and 4 (Table 2) such that Day 1 (Day 2) was used for training in Index 3 (Index 4) and Day 2 (Day 1) was used for testing.

Bold values indicate the accuracy of the full model.

## 4. Discussion

The long-term goal of our work is to build deployable BCI that predicts operator task and workload. This current project examined performance on data from varying degrees of similarity of collection, ranging from the training set to held-out data from a separate day of data collection. This allowed us to get a sense of the bias-variance trade-off space for predicting multi-task performance. For these analyzes, we relied primarily on a well-known ML modeling approach, support vector machines. The SVMs were able to learn consistent patterns of brain activity to predict two types of task demands: the number of tasks (one, two, or three), and the types of attentional resources demands (tracking, communicating, monitoring). To better understand the influence of the data used and compare with previously published work connecting EEG signals to task environments, we examined different subsets of the potential electrode-bandwidth components (320). We compared the accuracy of each electrode-frequency bandwidth subset to inform ML model predictions, at the individual-level. We then computed the loss in prediction accuracy should one attempt to generalize the model to classify unseen within- and between-session brain activity. As a follow-up analysis, we repeated our approach using subsets of the data corresponding to nine different commercial mobile EEG-systems which included fewer electrodes.

In all models, classifier decision bounds were set to maximally separate the electrode-bandwidth features between seven categories: T, C, M, T+C, T+M, C+M, and T+C+M. We assessed classifier performance relative to chance, which was one in seven or 14.3% accuracy. As expected, we found classifier accuracy depended on the number of features within each subset such that accuracy improved as more features were included. Additionally, we tested the reduction in classifier accuracy as we increased the level of generalizabilty of the testing dataset. The level of generalizabilty was higher for the between- compared to within-session classifications since it was further removed from the model it was trained on. Our results indicated that classifier accuracy decreased as the level of generalizability increased. For all subsets, model accuracy was the highest when classifying the subset of neural data it was trained on, followed by within- and between-session classifications, respectively.

The best performance was based on the full data set, including all bandwidths and electrodes. It may seem unsurprising to find that the best performing model is the one with the most features. Nonetheless, practitioners are limited by the electrode montage they can collect and the quality of the data at each electrode site. Our work investigated the accuracy of 19 unique subsets of the full model: 10 proposed in the literature (Model 2–11) and 9 montages of existing commercial devices (Model 12–20). Through this work, we aim to offer a practical guide under the necessary tradeoff when using mobile systems or subset of the full feature space. As such, the next best was a model based on only gamma activity across all 64 electrodes. This was consistent across all levels of generalizability; accuracy on the training set was 99.32% (full) and 89.66% (gamma), on new data within-session was 84.49% (full) and 82.77% (gamma), and on new data between-session was 32.01% (full) and 27.63% (gamma), where performance consistently remained well above chance (14.3%).

It is possible that at least some of the predictive strength associated with gamma activity is related to motor control. During the preparation and control of voluntary movement, gamma is thought to reflect the integration of sensory and motor information. Background gamma activity occurs even at rest due to thalomocortical loop activity, but motor movements increase gamma power spectral density above and beyond this low level. The topographical distribution of increases in gamma power has been leveraged to classify the type of motor command that was initiated. While single trial movement classification (type and lateralization) is often achieved using mu or beta rhythms (Kim et al., 2003; Alomari et al., 2013), power in the gamma frequency band is also informative. Amo et al. (2016) compared gamma power across left and right wrist movements and found variations as a function of handedness; the induced gamma response will be larger in a right handed individual moving their right wrist than when that same individual moves the left wrist (Amo et al., 2016). Gamma is also useful in decoding type of movement, even if those movements were simply imagined. In a single trial classification experiment of four different types of imagined right wrist movement, 66% of useful features were from the gamma band, found generally across an electrode montage for a 64-channel system (Vuckovic and Sepulveda, 2006). Our work demonstrates that variation in gamma power is informative for distinguishing task load and type in MAT-B tasks, and this could be attributed to the differences in motor requirements across conditions.

However, gamma activity is also associated with higher level cognitive processes that go beyond the motor demands of a particular task (Fitzgibbon et al., 2004). A previous investigation of the MAT-B task held task number and type constant, but changed difficulty mid trial (Bowers et al., 2014). These authors investigated changes in power in various frequency bands at multiple electrode sites in response to this change in difficulty. They found higher gamma power at temporal electrode sites during high difficulty. Runs that changed from easy to hard showed a clear increase in gamma power time locked to the transition point, and vice versa. However, the change in power was abrupt when difficulty was changed from easy to hard, and transitioned more gently when difficulty was changed from hard to easy. While difficulty changes may be associated with a change in the tempo of movements, gamma power changes were present despite no motor requirements being added or removed. Further, in a study using brain activity to classify single trial data from tasks with varying workload levels and sensory modalities, gamma band features were found to be the most reliably predictable across participants (Caywood et al., 2017). The importance of gamma features in the present study is likely due to a combination of changes to motor demands, difficulty level, and sensory modality.

Although beta power did not yield model accuracy quite as high as gamma power, it remained a practically useful predictor of task (i.e., 69.4% accurate in within-day testing) compared to theta and/or alpha power. Interestingly, previous work found beta power accurately predicted conditions differing in working memory demands (i.e., the n-back task; Ke et al., 2021), and, similarly, alpha power predicted the maintenance/exclusion of items in memory (Capilla et al., 2014) and exclusion of interfering items from memory (Rihs et al., 2007). However, we found higher model accuracy using beta compared to alpha power. The nature of the MATB tasks place very few explicit demands on working memory, and the dissociation we found in model accuracy suggests beta activity may capture changes in task demands beyond those of working memory alone, rather demands that are specific to the nature of the task(s).

We found models trained on alpha or theta activity performed moderately well when predicting (multi-) task condition, yielding around 49.5% accuracy in within-day testing. Nonetheless, performance suffers when compared to gamma or beta activity. Numerous manipulations to (mulit-)task demands many influence mental workload and neural activity, and alpha and theta power have primarily been investigated in the context of a simultaneous change in the level of difficulty for the entire environment (i.e., all tasks at once), rather than the addition (subtraction) of task(s), or the degree of task similarity where tasks compete for more (less) common resources. In our work, we manipulated the task (T, M, or C), the number of tasks (1, 2, or 3), and the degree of competition for common resources (TC, TM, and CM), and found evidence our demand manipulations may evoke different patterns of neural activity than that observed when all tasks change in their demands simultaneously. Hence, our work suggests that which neural indices are more informative to measure mental workload may depend on the type of demand manipulation the researcher is interested to predict. Neuroadaptive modeling may benefit from the development of a joint modeling approach to predict both the overall system demands and the task(s) one is performing at any given time. One can imagine leveraging several bandwidths to predict shifts in the task(s) being performed or a change in the demands of all tasks simultaneously.

One subset that performed particularly poorly was Set #8, the ratio proposed by Pope et al. (1995), where our results indicated the classifier accuracy to predict between-day data was not above chance level. This pattern indicated overfitting; we saw a large loss in model accuracy between classifications of the training dataset (our control; 61.82%) vs. new brain activity that were collected on the same day (19.78%), a substantial loss of about 42% accuracy resulting in only slightly better than chance performance (14.3%). Previous research has used the beta/(theta+alpha) ratio as a marker for workload. However, it should be noted that the ratio, and its variants, are used in many other studies, such as a marker of Attention Deficit/Hyperactivity Disorder (ADHD), risk-taking behavior, or resting, making the literature on it difficult to parse. Hence, it is not surprising that our results further indicate that a simple formula that takes the ratio of a few spectral bands cannot make better than chance level predictions without considering mediating factors (e.g., age or psychiatric conditions).

The ultimate goal of deploying an AI-enabled system is to augment, assist, or automate the environment or user in order to enhance overall HMT performance. We argue that BCI equipped to assess a psychological construct, such as mental workload, alone will not provide clear information regarding *how* to intervene in the most appropriate way. Further, the cause of current cognitive state and the decision on how to best complement the HMT highly depends on the task(s) and strategy of the user. In designing an AI-enabled system, it would be useful to not only understand when to intervene (e.g., workload is high) but also what task(s) the user is currently engaging in so that the system may better adapt for the user, given the context and their focus of attention.

In field applications, one is forced to trade-off between focusing on model specificity vs. implementation. We explored this space using one complex and multi-tasking environment, MAT-B, to offer a general prescriptive set of guidelines for predicting how many, and which, tasks one is completing at a given time. We found models trained from device subsets with fewer electrodes were outperformed by those which included a higher density array. Models trained using electrodes mapping to the Emotiv Epoc + system, which included only 14 electrodes, performed only marginally worse than those trained using electrodes corresponding to the layout of the Cognionics Quick Cap 20 (19 electrodes map to the 10-20 system) and the Emotiv Epoc Flex (32 electrodes). These results may help inform decisions about what methodological approach to emphasize, given goals of accuracy, generalizability, and practical feasibility such as physical constraints of the environment (e.g., in-flight) or other user equipment (e.g., headphones, helmet).

In our work, most models surpass the chance level for this 7-classes problem, but one may have a tolerance criterion for the minimum acceptable model performance. For example, nearly all models, including the full model, dropped below 30% accuracy when predicting an individual's data collected in a separate session. While it was better than chance, further model development is required for it to be useful in practice. Our work serves as a baseline recommendation for which mobile system may suffice for training a model to make multi-class decisions given their requirements for model accuracy. Additionally, these results give a sense of how well model settings can generalize across days and whether this degree of complexity requires a multi-dimensional array of electrodes and bandwidths. These types of investigations can improve our understanding of the connections between neural classification methods; it can also improve system design and boost the success rate of choosing the appropriate classification models to implement in environments that require mobility and versatility.

### 4.1. Future research

We formed a relatively comprehensive list of ways to test the generalizability of classification models (Table 6). For the research reported here, we choose to focus on models that both were trained and tested on an individual's data, for a particular day (i.e., Index 1 and Index 2) and models that were trained on an individual's data, for a particular day and was tested on the same individual's data from a different day (i.e., Index 3 and Index 4). Note that each of these 4 model types were run for all subjects (N), 2 demand manipulations, (T) and 11 neural input feature sets (F); we created and evaluated the performance of SVMs in 4 of these models. In future research, we plan to use Table 6 to inform how we may further systematically assess model generalizability.

**Table 6:** A systematic way to test the generalizability of classification models.

	**Training data**		**# of Classifiers**	**Testing data**		**Total # of Tests**
**Index**	**Level**	**Day**		**Level**	**Day**	
*1*	*Individual*	*Day 1*	*N*×*F* = 220	*Individual*	*Day 1*	*N*×*F* = 220
*2*	*Individual*	*Day 2*	*N*×*F* = 220	*Individual*	*Day 2*	*N*×*F* = 220
3	*Individual*	*Day 1*	*N*×*F* = 220	*Individual*	*Day 2*	*N*×*F* = 220
*4*	*Individual*	*Day 2*	*N*×*F* = 220	*Individual*	*Day 1*	*N*×*F* = 220
5	Individual	Both	*N*×*F* = 220	Individual	Day 1	*N*×*F* = 220
6	Individual	Both	*N*×*F* = 220	Individual	Day 2	*N*×*F* = 220
7	Individual	Both	*N*×*F* = 220	Individual	Both	*N*×*F* = 220
8	Group	Day 1	*F* = 11	Individual	Day 1	*N*×*F* = 220
9	Group	Day 2	*F* = 11	Individual	Day 2	*N*×*F* = 220
10	Group	Both	*F* = 11	Individual	Day 1	*N*×*F* = 220
11	Group	Both	*F* = 11	Individual	Day 2	*N*×*F* = 220
12	Group	Both	*F* = 11	Group	Day 1	*F* = 11
13	Group	Both	*F* = 11	Group	Day 1	*F* = 11
14	Group	Both	*F* = 11	Group	Day 1	*F* = 11

The current research involved Index 1–4 (italicized). The subset of data we used for training and testing was modeled at the individual-level and varied in whether it was randomly drawn from the Day 1 or Day 2. *N* = 20 (assuming no participants are excluded), F = number of input neural feature sets (11).

Importantly, our exploratory analyzes provided results from subsets of electrodes from our 64-channel dataset collected using a Biosemi ActiveTwo system in a controlled experimental setting. The real word applicability does not depend only on the number of electrodes of the commercial EEG systems considered. It also depends on the reliability of the EEG signal recorded by such devices. Future research should replicate these analyzes using the commercial device of interest.

Another consideration is that our models were static and unchanging. We did not incorporate any model learning or adaptation across time. In future work, we could adapt our model using new data, as long as those data have a *ground-truth* of the correct classification. This would hopefully reduce the impact of overfitting and hence improve model generalizability. Repeatedly introducing the model to more sources of noise may offer a type of learning that is flexible to account for idiosyncrasies that may be highly characteristic of one's brain activity. In turn, these models will more accurately capture true variation driven by condition (e.g., task type, degree of cognitive load).

In a similar way, we can train models using the brain activity of multiple people, variations of broad-level types of tasks (e.g., MATB tracking and aircraft simulator), and several different environmentally imposed constraints (communications in silence and embedded within cockpit noise). Certainly variations in neural activity exist as models become more generalized, and some recent work in this domain shows initial promise through model transfer to new tasks (Gupta et al., 2021) and people (Zhou et al., 2022) when classifying a low or high level of cognitive workload. Understanding which neural features are consistent and differentiate among several broad-level task categories will maximize model accuracy in generalizing to new contexts. In addition, this future work will improve our theoretical understanding of how neural activity and cognitive processing may depend on cognitive load and the nature of the task(s) being performed (e.g., tracking, monitoring, communications).

## 5. Conclusions

Our work systematically investigated a major challenge in neuroergonomics, the ability to assess and predict an operators activity and workload. Through a carefully controlled experiment and analytic framework, we identified the extent that features of neural activity and a machine learning approach may provide generalizable predictions across multi(task) contexts and sessions. We trained and tested SVM models using individual's neural data to classify the number and type of tasks being completed. We compared the accuracy of 10 electrode-frequency bandwidth subsets and nine commercial EEG systems across three levels of generalizability. We found model accuracy improved as more features were included and decreased as a function of generalized predictions (within- followed by between-session). We found gamma activity performed nearly as well as a full model, which may in part be attributed to both its relationship with motor activity and workload demands. In addition, we found that despite having fewer electrodes some commercial systems may offer the neural features necessary to obtain a fairly high level of accuracy compared to a higher density system. Our research offers a novel and practical modeling solution to predict task and cognitive load through brain activity. In addition, we provide estimates for system designers and researchers to make informed decisions about if (how) to generalize models of neural activity to predict (multi)task and cognitive workload.

## Data availability statement

The raw data supporting the conclusions of this article will be made available by the authors, without undue reservation. The study data/code is available at Open Science Foundation: https://osf.io/8kc7w/.

## Ethics statement

The studies involving human participants were reviewed and approved by Wright State University. The patients/participants provided their written informed consent to participate in this study. Permission has been obtained from the individuals to use their images in the publication.

## Author contributions

EF and JH contributed to conception and design of the study. EF organized the database and performed the statistical analysis. All authors wrote sections of the manuscript, contributed to manuscript revision, read, and approved the submitted version.

## Funding

The research was supported, in part, by the 711 Human Performance Wing Chief Scientist Office (Contract # FA8650-20-D-6203).

## Conflict of interest

The authors declare that the research was conducted in the absence of any commercial or financial relationships that could be construed as a potential conflict of interest.

## Publisher's note

All claims expressed in this article are solely those of the authors and do not necessarily represent those of their affiliated organizations, or those of the publisher, the editors and the reviewers. Any product that may be evaluated in this article, or claim that may be made by its manufacturer, is not guaranteed or endorsed by the publisher.

## Author disclaimer

The views expressed are those of the author and do not necessarily reflect the official policy or position of the Department of the Air Force, the Department of Defense, or the United States Government. Distribution A: Approved for public release; distribution unlimited. AFRL-2022-3239; Cleared 08 July 2022.

## Footnotes

^1^The definition of Gamma activity varies between 30 and 100 Hz. Since high frequencies are typically contaminated with line noise (50–60 Hz) or muscle activity (70–100 Hz), we apply a lowpass filter to assess 1–40 Hz (Andreassi, 2013), where 25–40 Hz is sometimes referred to as the “low-gamma” band. Typically gamma activity related to cognitive functioning is associated with band-power between 25 and 55 Hz (e.g., Choi et al., 2018; Tremmel et al., 2019). Researchers should take note of this when considering gamma activity fluctuations in relation to our data or their own future work.
